# Preliminary *in vitro* evaluation of the nutritional characteristics of *Tithonia diversifolia* forage meal for pig diets

**DOI:** 10.14202/vetworld.2026.1484-1494

**Published:** 2026-04-24

**Authors:** Jose Alberto de la Torres Moreira, Verónica Cristina Andrade Yucailla, Johana Elizabeth Delgado Lozada, Milton Andres Montalvo Lozada, Raciel Lima Orozco

**Affiliations:** 1 Fauna, Conservation and Global Health Research Group – Universidad Regional Amazónica Ikiam, Tena, Ecuador; 2 Centro de Investigaciones Agropecuarias, Facultad de Ciencias Agropecuarias, Universidad Estatal Península de Santa Elena, La Libertad, Ecuador; 3 Departamento de Medicina Veterinaria, Universidad Central “Marta Abreu” de Las Villas, Santa Clara, Cuba; 4 Centro de Investigaciones Agropecuarias, Universidad Central “Marta Abreu” de Las Villas, Santa Clara, Cuba

**Keywords:** amino acid profile, *in vitro* digestibility, monogastric nutrition, net energy estimation, non-conventional forage, pig diets, sustainable feed resources, *Tithonia diversifolia*

## Abstract

**Background and Aim:**

The increasing cost of conventional feed ingredients and the demand for sustainable alternatives have intensified the search for unconventional forage resources in pig nutrition. *Tithonia diversifolia* is a fast-growing tropical shrub with high biomass yield and promising nutritional composition. However, its application in monogastric feeding systems remains insufficiently characterized. This study aimed to evaluate the chemical composition, amino acid profile, and *in vitro* digestibility of *T. diversifolia* forage meal at graded inclusion levels in pig diets.

**Materials and Methods:**

Forage samples of *T. diversifolia* were harvested in Napo, Ecuador, processed into meal, and subjected to proximate and amino acid analyses. Five experimental diets containing 0%, 10%, 15%, 20%, and 25% forage meal inclusion were formulated. Chemical composition and *in vitro* digestibility were assessed with four replicates per treatment. Digestible energy, metabolizable energy (ME) for growing pigs, ME for finishing pigs, and net energy were estimated using established regression equations. Statistical analysis was performed using one-way analysis of variance followed by Tukey’s post hoc test, with significance declared at p < 0.05.

**Results:**

*T. diversifolia* meal exhibited high crude protein and mineral content but also elevated fiber and lignin levels. The amino acid profile showed appreciable concentrations of leucine, valine, and lysine, whereas methionine and tryptophan were limited. Inclusion of *T. diversifolia* significantly reduced *in vitro* dry matter digestibility compared to the control diet (p < 0.05), although no significant differences were observed among inclusion levels from 10% to 25%. Crude protein and neutral detergent fiber content varied significantly among treatments (p < 0.05), while other proximate parameters remained stable. Estimated energy values did not differ significantly across diets (p > 0.05), despite a numerical decline with increasing inclusion levels.

**Conclusion:**

*T. diversifolia* forage meal demonstrates potential as a protein- and mineral-rich alternative ingredient in pig diets. Inclusion levels up to 25% maintained acceptable *in vitro* digestibility and energy values, although higher fiber and lignin contents may limit nutrient utilization at elevated levels. Moderate inclusion levels are recommended to balance nutritional benefits and digestibility constraints. Further *in vivo* studies are required to validate its practical application and effects on animal performance.

## INTRODUCTION

The increasing cost and competition for conventional feed ingredients in pig production systems have intensified the search for alternative and locally available protein sources, particularly in tropical regions [1, 2]. In this context, the use of fast-growing forages with high nutritional potential represents a viable strategy to reduce dependence on soybean meal and improve feed autonomy for both smallholder and commercial producers [3]. *Tithonia diversifolia* (Hemsl.) A. Gray is a perennial shrub widely distributed in tropical ecosystems, characterized by rapid biomass accumulation, adaptability to low-fertility soils, and a notable crude protein and mineral content, which has supported its use in ruminant feeding systems [4].

However, despite increasing agronomic interest, the nutritional characterization of *T. diversifolia* for monogastric animals remains limited. Existing studies have primarily focused on ruminant systems or partial proximate analyses, with limited information available on amino acid balance, digestible and metabolizable energy (ME) estimation, and graded responses in digestibility under simulated monogastric conditions [5–7]. Furthermore, there is no standardized dataset describing how varying inclusion levels of *T. diversifolia* influence the nutritional and energetic profile of formulated pig diets, thereby restricting its practical application in swine nutrition [8]. To the best of our knowledge, this is the first report describing the amino acid profile and *in vitro* energy estimation of *T. diversifolia* meal specifically formulated for pig diets under Amazonian Ecuadorian conditions.

Despite the recognized agronomic and nutritional potential of *T. diversifolia*, its application in monogastric nutrition remains inadequately explored. Most existing studies have focused on ruminant feeding systems, where the high-fiber content is better tolerated, while investigations in pig nutrition are limited and often restricted to basic proximate composition. Critically, there is a lack of comprehensive data integrating amino acid profiling, energy estimation, and digestibility responses under controlled *in vitro* conditions that simulate monogastric digestion. Furthermore, previous studies have not systematically evaluated graded inclusion levels of *T. diversifolia* within complete diets, resulting in insufficient evidence regarding optimal inclusion thresholds and their impact on nutrient utilization. The absence of standardized datasets linking chemical composition with functional nutritional outcomes, particularly under tropical production conditions, represents a significant limitation. This knowledge gap constrains the practical incorporation of *T. diversifolia* into pig diets and highlights the need for a holistic evaluation that combines compositional, energetic, and digestibility parameters.

Therefore, the present study was designed to provide a comprehensive evaluation of *T. diversifolia* forage meal as a potential alternative ingredient in pig nutrition. Specifically, the objectives were to (i) characterize the proximate composition, mineral content, and amino acid profile of *T. diversifolia* meal; (ii) assess the effects of increasing inclusion levels (0%, 10%, 15%, 20%, and 25%) on the chemical composition and *in vitro* digestibility of formulated pig diets; and (iii) estimate digestible energy, ME for growing pigs, ME for finishing pigs, and net energy (NE) using established predictive models. By integrating compositional and functional analyses, this study aims to generate evidence-based insights into the nutritional value and feasible inclusion levels of *T. diversifolia*, thereby supporting its application as a sustainable feed resource in tropical pig production systems.

## MATERIALS AND METHODS

### Ethical approval

This study was conducted in accordance with internationally accepted ethical standards for research and reporting. All procedures complied with the ARRIVE 2.0 guidelines for transparent reporting of scientific studies and adhered to the principles outlined in the EU Directive 2010/63/EU on the protection of animals used for scientific purposes. Ethical clearance was obtained from the Ethical Committee of the Scientific Council, Faculty of Agricultural Sciences, Universidad Central “Marta Abreu” de Las Villas, Cuba (Protocol No. 57/2021; approved on March 14, 2021).

The experimental design involved exclusively *in vitro* analyses of plant-based feed materials, and therefore no live animals were subjected to experimental procedures, handling, or invasive sampling. Consequently, no animal welfare risks were identified. All laboratory procedures were conducted under standard biosafety conditions in accordance with institutional laboratory safety protocols.

Plant material (*T. diversifolia*) was collected from agricultural land in Napo Province, Ecuador, with full compliance to national agricultural and environmental regulations. The collection did not involve protected species or restricted areas, and no specific permits were required under local legislation. Handling, processing, and disposal of plant materials followed established environmental safety guidelines to minimize ecological impact.

The study design, data handling, and reporting were conducted in accordance with principles of scientific integrity, reproducibility, and transparency. No human participants or personal data were involved in this study.

### Study period and location

The study was conducted from March to July 2025 in Napo Province, Tena Canton, Misahuallí Parish (Santo Urku community), Ecuador (0°57′01″S; 77°51′46″W; 565 m a.s.l.). The region has a humid tropical climate (Af, Köppen classification), with an annual rainfall of approximately 3800 mm, a mean temperature of 24°C–26°C, and relative humidity of around 85%. Soils are sandy loam, acidic (pH 4.5–5.5), and of moderate fertility.

*In vitro* digestibility tests were performed at the Natural Products Laboratory, Universidad Regional Amazónica Ikiam (Tena, Ecuador). Harvesting was carried out at a cutting height of 50 cm from the ground [9], and the evaluation of green and dry matter production was conducted using the method proposed by Rodríguez and Torres [10].

### Chemical composition

The bromatological characterization of the meal was conducted using 2 kg samples of green forage, analyzed in triplicate for each treatment. Samples were dried using a vertical solar dryer at 55°C ± 2°C for 72 h until reaching approximately 10% residual moisture. The dried material was ground using a Willey mill (Thomas Scientific, USA) with a 1 mm sieve, homogenized, and stored in airtight polyethylene bags for subsequent analyses.

Proximate analysis was performed according to U. Florida [11, 12], including dry matter (DM), organic matter (OM), crude protein (CP; Kjeldahl method, N × 6.25), ether extract (EE), and nitrogen-free extract (NFE). Fiber fractionation (neutral detergent fiber [NDF] and acid detergent fiber [ADF]) was determined according to Van Soest *et al*. [13]. Additionally, analyses followed AOAC International (2016) methods: DM (934.01), CP (976.05), EE (920.39), and ash (942.05). Lignin content was determined using permanganate oxidation.

Gross energy (GE), ME, and digestible energy (DE) were estimated using predictive equations based on nutritional composition (Table 1) [14, 15]. Mineral composition, including phosphorus (P), potassium (K), calcium (Ca), magnesium (Mg), iron (Fe), copper (Cu), zinc (Zn), and manganese (Mn), was analyzed using atomic absorption spectrophotometry [16].

**Table 1:** Equations used to estimate gross energy [14], digestible energy [15], and metabolizable energy [16] of *Tithonia diversifolia* based on its nutritional composition.

Energy	Equation
Gross energy (GE)	GE (kcal/kg DM) = (5.7 × CP) + (9.4 × EE) + (4.1 × NFE) + (4.1 × Fiber)
Digestible energy (DE)	DE (kcal/kg DM) = 100.5 − (0.079 × Ash) − (0.088 × NDF) − (0.11 × Lignin)
Metabolizable energy (ME)	ME (kcal/kg MS) = ED (kcal/kg MS) × 0.82

CP = Crude protein, EE = Ether extract, NDF = Neutral detergent fiber, ADF = Acid detergent fiber, NFE = Nitrogen-free extract.

Sixteen amino acids were quantified by high-performance liquid chromatography (HPLC; Agilent 1100 system, Ecuachemlab Cía. Ltda., Ecuador) following acid hydrolysis (6 N HCl, 110°C, 24 h). Tryptophan was analyzed separately using alkaline hydrolysis with fluorescence detection. Data were expressed as percentage of sample dry weight (% w/w).

### *In vitro* digestibility of *T. diversifolia*

The study was conducted at the Natural Products Laboratory of Universidad Regional Amazónica Ikiam using five treatments based on formulated diets containing different inclusion levels of *T. diversifolia* meal (10%, 15%, 20%, and 25%), along with a control treatment (0%). Each treatment was evaluated with four replicates under a completely randomized design.

Proximate chemical composition parameters of all treatments (DM, EE, CP, and OM) were determined (Table 2). Based on *in vitro* OM digestibility values, DE and NE were estimated using predictive equations described by [17]. In addition, ME for growing pigs and finishing pigs was calculated according to the models proposed by previous studies [18, 19] (Table 3).

**Table 2:** Chemical composition of *Tithonia diversifolia* flour.

Parameter	g/kg (DM)
Crude protein	318.127
Ether extract	10.444
Crude fiber	366.146
Ash	176.471
Nitrogen-free extracts	127.731
Neutral detergent fiber	534.214
Acid detergent fiber	533.013
Detergent lignin	292.917
Phosphorus	7.203
Potassium	39.616
Calcium	32.413
Magnesium	4.682
Iron	0.267
Copper	0.003
Zinc	0.113
Manganese	0.068

**Table 3:** Regression equations used in the study to estimate the *in vivo* digestibility of organic matter (dMO), digestible energy (DE), metabolizable energy for growing pigs (EMcr) and pigs finishing fattening (EMfc) according to Noblet and Shi [19].

Equation	R²
dMO = 0.409 + 0.608 dvMO − 0.00063 NDF − 0.00061 Ash	0.90
DE = 6.05 + 0.0116 dvMO + 0.0166 EE − 0.0135 ADF	0.88
MEcr = ED × (1.012 − (0.0019 × CP))	0.91
MEfc = 1107 + (0.64 × MEcr) + (22.9 × EE) + (6.9 × CP)	0.96
NE = 3.22 + 0.0072 dvMO + 0.0039 St + 0.0197 EE − 0.0109 ADF	0.94

NDF = Neutral detergent fiber, ADF = Acid detergent fiber, EE = Ether extract, CP = Crude protein, St = Starch, dvMO = *in vitro* digestibility of organic matter, DE = Digestible energy.

Five experimental diets were formulated containing 0%, 10%, 15%, 20%, and 25% *T. diversifolia* meal as a partial replacement for soybean meal and fish meal. The control (basal) diet was formulated using corn meal, soybean meal, fish meal, wheat bran, and a vitamin–mineral premix (Table 4). All diets were designed to be iso-nitrogenous and iso-energetic, meeting the nutrient requirements for growing pigs according to NRC (National Research Council, 2012) [20].

**Table 4:** Ingredient and nutrient composition of the basal (control) diet for growing pigs (g/kg DM).

Ingredient	g/kg DM
Corn meal	600
Soybean meal	220
Fish meal	100
Vitamin–mineral premix	30
Wheat bran	50
Total	1000

Crude protein: 174 g/kg DM, Metabolizable energy: 14.2 MJ/kg DM, Ether extract: 35 g/kg DM, Crude fiber: 42 g/kg DM. DM = Dry matter.

### Statistical analysis

Statistical analyses were performed using R software version 4.3.1 (R Core Team, 2023, Vienna, Austria) under a completely randomized design with one-way analysis of variance. Data are expressed as mean ± standard deviation, and significance was declared at p < 0.05 using Tukey’s post hoc test.

## RESULTS

### Chemical composition of *T. diversifolia* flour

*T. diversifolia* flour had a CP content of 318.127 (g/kg DM), with high levels of crude fiber (366.146 g/kg DM) and ash (176.471 g/kg DM). EE content was low (10.444 g/kg DM), while the neutral detergent fiber (NDF) and ADF contents were 534.214 (g/kg DM) and 533.013 (g/kg DM), respectively. Lignin reached 292.917 (g/kg DM), indicating a structurally complex fibrous fraction. In addition, the sample had appreciable levels of minerals such as calcium (32.413 g/kg DM), potassium (39.616 g/kg DM), and phosphorus (7.203 g/kg DM).

### Amino acid profile

**Figure 1:**
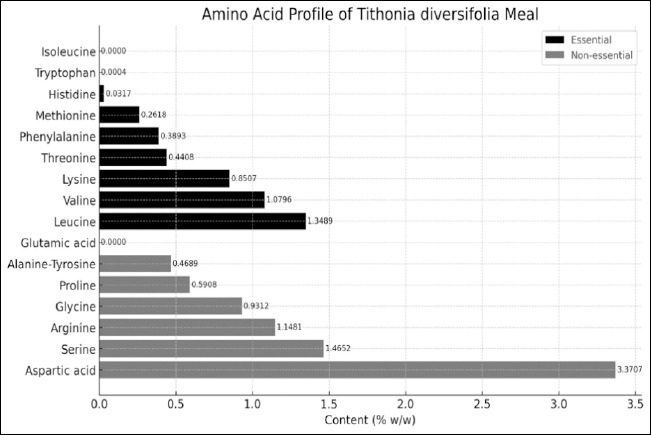
Amino acid profile of *Tithonia diversifolia* flour. The figure shows the percentage content of 16 amino acids determined by high-performance liquid chromatography, expressed as a percentage of wet weight (% w/w). Essential amino acids are represented by black bars, while nonessential amino acids are represented by gray bars.

Chromatographic analysis revealed a total amino acid concentration of 12.38% (Figure 1). The most abundant amino acids were aspartic acid (3.37%), serine (1.46%), leucine (1.35%), valine (1.08%), and glycine (0.93%). Significant amounts of lysine (0.85%) were detected, whereas methionine was low (0.26%). Tryptophan was detected in minimal concentrations (0.0004%), and neither isoleucine nor glutamic acid were detected.

### *In vitro* digestibility and chemical composition of experimental diets

The results of the chemical analysis (Table 5) of experimental diets with different levels of *T. diversifolia* flour inclusion showed no statistically significant differences (p > 0.05) in DM, OM, EE, or ADF contents among treatments. However, significant differences were observed in CP content (p = 0.03) and NDF content (p = 0.02).

**Table 5:** Proximate chemical analysis: dry matter (DM, g/kg as feed); organic matter (OM), crude protein (CP), ether extract (EE), starch (St), neutral detergent fiber (NDF) and acid detergent fiber (ADF) (g/kg DM) of the studied treatments and of the *Tithonia diversifolia* flours (n = 5).

Treatments (T)	DM		OM		CP		EE		ST		NDF		ADF	

	x̄	SD	x̄	SD	x̄	SD	x̄	SD	x̄	SD	x̄	SD	x̄	SD
Control	894.98	3.64	917.03	3.92	173.90^ab^	4.02	30.75	1.53	398.78	13.91	192.45^ab^	5.27	78.00	5.65
T1 (10 %)	889.63	5.62	919.78	4.80	174.38^ab^	1.86	30.65	1.77	400.03	8.60	196.63^ab^	6.08	78.75	4.62
T2 (15 %)	893.60	6.00	917.08	5.78	176.20^ab^	3.40	31.03	2.64	401.93	13.29	188.78^a^	1.85	74.35	3.38
T3 (20 %)	890.30	4.80	923.95	8.55	178.38^b^	1.60	31.38	2.27	405.88	5.57	201.23^b^	2.55	76.45	5.31
T4 (25 %)	890.85	1.66	917.65	4.91	171.68^a^	1.26	29.98	1.92	390.83	9.66	197.13^ab^	6.37	79.60	3.91
p-value	0.4416		0.4284		**0.03**		0.9033		0.4019		0.02		0.5461	

The values refer only to treatments studied according to analysis of variance (analysis of variance). Values are expressed as mean (X) ± standard deviation (SD). Control: 100 % feed (finishing pig concentrate), T1: 90 % feed + 10 % *T. diversifolia*, T2: 85 % feed + 15 % *T. diversifolia* flour, T3: 80 % feed + 20 % *T. diversifolia*, T4: 75 % feed + 25 % *T. diversifolia* flour. Identical superscript letters within the same column did not differ statistically (p > 0.05) according to Tukey test. Different superscript letters within the same column differed statistically (p < 0.05) according to Tukey test.

Crude protein content showed a progressive increase from the control treatment (173.9 g/kg DM) to treatment T3 (20%), reaching a maximum value of 178.38 g/kg DM, followed by a decrease in T4 (171.68 g/kg DM). Post hoc analysis indicated that T3 differed significantly from the treatment with the highest inclusion level (T4), whereas treatments T1, T2, and the control exhibited intermediate values.

In terms of NDF, treatment T3 (20%) showed the highest value (201.23 g/kg DM), which was significantly higher than T2 (15%) with 188.78 g/kg DM. The remaining treatments did not show significant differences among them.

### Starch, proximate stability, and energy trends

Total starch content (ST) ranged from 390.83 g/kg DM (T4) to 405.88 g/kg DM (T3), with no statistical differences (p = 0.4019). EE values remained constant (29.98–31.38 g/kg DM), with no evidence of treatment effects (p = 0.9033). DM and OM also remained stable among treatments (p > 0.4), indicating that the inclusion of *T. diversifolia* meal up to 25% does not significantly affect these fractions.

**Figure 2:**
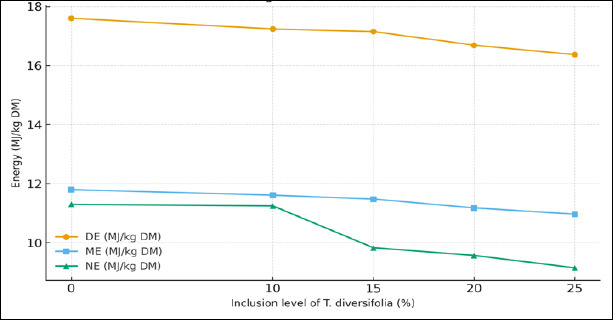
*In vitro* energy values (DE= digestible energy, ME= metabolizable energy, and NE= net energy; MJ/kg DM) of diets with increasing inclusion levels of *Tithonia diversifolia* forage meal. Data represent mean values of five inclusion treatments (T0 = 0 %, T1 = 10 %, T2 = 15 %, T3 = 20 %, and T4 = 25 %).

Energy trends derived from the *in vitro* digestibility assays (Figure 2) indicated a gradual numerical decline in DE, ME, and NE values as the inclusion level of *T. diversifolia* increased from 0% to 25%.

### *In vitro* digestibility and energy estimation

The *in vitro* digestibility of DM and OM showed a numerical reduction with increasing levels of *T. diversifolia* inclusion (Table 6). However, no significant differences (p > 0.05) were observed among the 10%–25% inclusion treatments, indicating that the reduction pattern was not strictly progressive. The control diet (0%) exhibited slightly higher digestibility coefficients, followed by minor decreases in the experimental diets, although the overall variation remained within an acceptable range.

**Table 6:** Analysis of *in vitro* digestibility of organic matter (dvOM) and estimates of *in vivo* digestibility of organic matter (dOM), digestible energy (DE), metabolizable energy for growing pigs (MEcr) and finishing fattening pigs (MEfc) and net energy (NE) of the treatments studied (n=5).

Treatments (T)	dvDM		dvOM		dOM		DE		MEcr		MEfc		NE	

	X	SD	X	SD	X	SD	X	SD	X	SD	X	SD	X	SD
Control	42.88^b^	3.13	928.08^a^	35.4	564.24^a^	21.53	16.83^a^	0.41	11.28^a^	0.28	12.58^a^	0.18	9.44^a^	0.25
T1 (10 %)	36.88^ab^	4.91	940.76^a^	51.34	571.95^a^	31.22	16.98^a^	0.60	11.37^a^	0.40	12.58^a^	0.26	9.53^a^	0.37
T2 (15 %)	36.39^ab^	4.62	924.40^a^	1.53	562.10^a^	0.98	16.79^a^	0.02	11.25^a^	0.01	12.58^a^	0.01	9.41^a^	0.01
T3 (20 %)	31.68^a^	2.72	940.16^a^	21.64	571.59^a^	13.16	16.97^a^	0.25	11.37^a^	0.17	12.58^a^	0.11	9.53^a^	0.16
T4 (25 %)	36.71^ab^	2.52	898.44^a^	8.65	546.22^a^	5.26	16.49^a^	0.10	11.05^a^	0.07	12.58^a^	0.04	9.23^a^	0.06
p-value	0.0128		0.4509		0.4512		0.4509		0.4509		0.4509		0.4509	

The values refer only to treatments studied according to analysis of variance (analysis of variance). Values are expressed as mean (X) ± standard deviation (SD). Control: 100 % feed (finishing pig concentrate), T1: 90 % feed + 10 % *Tithonia diversifolia*, T2: 85 % feed + 15 % *T. diversifolia* flour, T3: 80 % feed + 20 % *T. diversifolia*, T4: 75 % feed + 25 % *T. diversifolia* flour. Identical superscript letters within the same column did not differ statistically (p > 0.05) according to Tukey test. Different superscript letters within the same column differed statistically (p < 0.05) according to Tukey test.

Correspondingly, the estimated energy values (DE, ME, and NE) did not differ statistically (p > 0.05) among treatments, although a mild numerical downward trend was observed with increasing inclusion levels. These findings suggest that *T. diversifolia* meal can be incorporated up to 25% in pig diets without significantly compromising *in vitro* digestibility or energy content.

## DISCUSION

### Nutritional composition of *T. diversifolia* flour

*T. diversifolia* flour has a nutritional profile that stands out for its high CP content (318.127 g/kg DM), making it a promising alternative in animal diets, especially in production systems seeking accessible and sustainable protein sources. This value falls within the range reported by various authors, who have documented concentrations between 264.100 g/kg DM and 348.100 g/kg DM depending on factors such as the physiological age of the plant, cutting frequency, and agroecological conditions [21].

The crude fiber content was high (366.146 g/kg DM), as were the NDF (534.214 g/kg DM) and ADF (533.013 g/kg DM) values, reflecting a considerable structural fraction. These values suggest limited DM digestibility, especially for monogastric animals, although they could be functional as effective fiber in ruminants [4]. Lignin reached 292.917 (g/kg DM), a value that compromises the digestibility of the fibrous fraction by forming lignocellulosic complexes that are resistant to enzymatic action [22]. It has been observed that lignin levels above 180.100 (g/kg DM) can significantly reduce nutrient degradability in monogastric animals [23], and therefore this value must be carefully considered when formulating diets.

The low EE content (10.444 g/kg DM) is characteristic of shrubby forages, limiting their energy contribution via lipids. However, the energy balance can be maintained by including other energy-rich ingredients in the diet [24]. Ash accounted for 176.471 (g/kg DM), indicating significant mineral richness. This value is often associated with the presence of essential macro- and microminerals, as evidenced by the levels of calcium (32.413 g/kg DM), potassium (39.616 g/kg DM), and phosphorus (7.203 g/kg DM). The calcium content is particularly relevant for the diets of growing and lactating animals [25], while potassium and phosphorus play fundamental roles in osmotic regulation and energy metabolism [26].

The magnesium level (4.682 g/kg DM) and values for trace elements such as iron (0.267 g/kg DM), zinc (0.113 g/kg DM), manganese (0.068 g/kg DM), and copper (0.003 g/kg DM) are adequate and comparable to other tropical forage species [27]. The bioavailability of these elements must be evaluated based on their interaction with other dietary components, especially fiber and phytates [28]. Although the copper content was low, it can be supplemented with external sources according to the requirements of the animal species [29].

Overall, the nutritional profile of *T. diversifolia* flour indicates interesting potential as a protein–mineral supplement in diets for ruminants and, to a lesser extent, for monogastric animals, provided that its high-fiber and lignin content is considered. Its use could be particularly suitable in silvopastoral systems or as a component of multinutritional mixtures [30]. However, further *in vivo* or *in vitro* digestibility studies are recommended to confirm its effective nutritional value, as well as acceptance and palatability tests in the target species [31].

### Amino acid profile

Chromatographic analysis of *T. diversifolia* flour revealed a total amino acid content of 12.38%, confirming its potential as a protein source for animal feed systems in tropical areas. This value significantly exceeds the contribution of conventional tropical grasses (Cenchrus clandestinum: 8–10% CP) and is comparable to the range reported in forage legumes such as Leucaena leucocephala (18%–25%) [5].

Among essential amino acids, leucine and valine predominated, with values of 1.35% and 1.08%, respectively. These amino acids are essential for muscle metabolism and protein synthesis, particularly in growing animals [32]. The concentration of lysine (0.85%) is also noteworthy, as it is commonly a limiting amino acid in plant-based diets, highlighting the potential of *T. diversifolia* as a complementary ingredient in monogastric diets [33].

However, low concentrations of methionine (0.26%) and an almost negligible presence of tryptophan (0.0004%) were observed, along with the absence of isoleucine and glutamic acid, which represents a nutritional limitation. The deficiency of methionine, an essential sulfur-containing amino acid, can compromise protein utilization efficiency [34]. Therefore, it is advisable to formulate diets that compensate for this deficiency, either through methionine-rich ingredients such as treated soybean meal or through synthetic supplementation [35].

Among non-essential amino acids, aspartic acid (3.37%), serine (1.46%), and glycine (0.93%) were predominant. These compounds play key roles as metabolic precursors and in the synthesis of nucleotides, collagen, and other structural components [36–38].

Overall, the amino acid profile suggests that *T. diversifolia* is a valuable resource for diet formulation; however, its use must be carefully balanced to address deficiencies in essential amino acids. Integration of this ingredient into animal diets should be supported by evaluations of true protein digestibility and productive performance, particularly in species sensitive to amino acid imbalances [39].

### *In vitro* digestibility and nutritional implications

The results of the chemical analysis (Table 5) indicate that the progressive inclusion of *T. diversifolia* flour up to 25% did not significantly affect the DM, OM, EE, or ADF contents (p > 0.05), which is consistent with previous findings [6]. This behavior suggests that the incorporation of this forage does not substantially alter the structural or non-DE fractions of the diet [40]. However, significant effects were observed for CP (p = 0.03) and NDF (p = 0.02), which are critical parameters in diet formulation.

CP content increased up to treatment T3 (20%), reaching 178.38 g/kg DM, followed by a decrease in T4 (25%). This pattern suggests that moderate inclusion levels may enhance protein intake, possibly due to the high concentration of soluble nitrogen in the leaves of this species. The reduction observed in T4 may be attributed to protein dilution associated with increased fiber content, a phenomenon also reported in diets with high inclusion of fibrous forages [41]. Post hoc analysis confirmed a significant difference between T3 and T4, indicating that 20% may represent an optimal inclusion level for maximizing protein content.

NDF content was highest in T3 (201.23 g/kg DM), significantly exceeding that of T2 (188.78 g/kg DM). This increase in effective fiber may contribute to satiety in monogastric animals and stimulate rumination in ruminants [42], although it may also reduce digestibility. Despite variations in CP and NDF, total starch content remained stable (p = 0.40), suggesting that non-structural carbohydrate fractions were not affected by *T. diversifolia* inclusion [43]. EE content also remained constant (p = 0.90), confirming the limited lipid contribution of this forage.

*In vitro* DM digestibility showed a significant decrease (p = 0.0128) with increasing inclusion levels, particularly in treatment T3 (31.68%) compared with the control (42.88%). Although no significant differences were observed in *in vitro* digestibility of OM or in estimated energy values (DE, MEcr, MEfc, and NE), a general numerical decline (p > 0.1) was evident as inclusion levels increased. This reduction may be associated with increased structural fiber and lignified fractions, which limit enzymatic degradation and nutrient availability [44]. According to Weimer [45], elevated lignin and hemicellulose levels reduce cell wall degradability and negatively impact digestive efficiency.

Despite this trend, treatments T1 (10%) and T2 (15%) maintained moderate energy losses and acceptable digestibility coefficients (>36%) (Table 6), indicating that low to moderate inclusion levels may be viable without significantly compromising nutritional value [46]. This finding is relevant for practical applications, as it supports the use of *T. diversifolia* as a functional and economical feed component without adversely affecting animal performance [47]. Although DE and NE values did not differ significantly (p > 0.45), the control diet maintained the highest values (16.83 kcal/kg and 9.44 kcal/kg, respectively), likely reflecting the higher efficiency of conventional diets lacking high-fiber components [48].

## CONCLUSION

The present study demonstrated that *T. diversifolia* forage meal possesses a nutritionally valuable profile characterized by high CP content (318.127 g/kg DM) and appreciable mineral levels, alongside a moderate amino acid composition dominated by leucine, valine, and lysine. However, the high-fiber fractions, particularly NDF (534.214 g/kg DM), ADF (533.013 g/kg DM), and lignin (292.917 g/kg DM), were identified as critical limiting factors affecting nutrient utilization. The *in vitro* evaluation revealed that increasing inclusion levels of *T. diversifolia* resulted in a significant reduction in DM digestibility (p < 0.05), while OM digestibility and estimated energy values (DE, ME, and NE) remained statistically unaffected (p > 0.05), despite a consistent numerical decline.

From a practical perspective, inclusion levels of 10%–15% appear to represent an optimal balance between nutritional contribution and digestibility, whereas higher inclusion levels (20%–25%) may compromise feed efficiency due to increased structural fiber and lignification. These findings suggest that *T. diversifolia* can be effectively utilized as a complementary protein–mineral source in pig diets, particularly in tropical production systems where conventional feed resources are limited or costly. Its incorporation may contribute to improved feed sustainability and reduced dependence on soybean-based ingredients.

A key strength of this study lies in its integrated approach combining chemical composition, amino acid profiling, and *in vitro* digestibility with energy estimation under controlled conditions. This provides a more comprehensive understanding of the functional nutritional value of *T. diversifolia* compared with studies limited to proximate analysis. Additionally, the evaluation of graded inclusion levels offers practical insights for feed formulation.

However, the study is limited by its reliance on *in vitro* methodologies, which may not fully replicate *in vivo* digestive and metabolic responses in pigs. Furthermore, factors such as palatability, anti-nutritional compounds, and amino acid bioavailability were not assessed, which may influence the practical applicability of the results.

Future research should focus on *in vivo* feeding trials to validate digestibility, growth performance, and health outcomes in pigs. Investigations into processing methods to reduce fiber and lignin content, as well as strategies to correct amino acid imbalances, are also recommended. Additionally, economic feasibility and long-term sustainability assessments will be essential to support large-scale application.

In conclusion, *T. diversifolia* forage meal represents a promising alternative feed resource with significant potential for sustainable pig production, provided that its inclusion is optimized to balance nutritional benefits and digestibility constraints.

## DATA AVAILABILITY

The data generated and analyzed during this study are included in this published article. Additional data are available from the corresponding author upon reasonable request.

## AUTHORS’ CONTRIBUTIONS

JADLTM: Conceptualization, study design, supervision, data analysis, and writing–original draft preparation, RLO: Study design, data interpretation, and critical revision of the manuscript, VCAY: Experimental design, data collection, and manuscript editing, JEDL: Sample processing, data collection, and manuscript revision, MAML: Laboratory analyses, data collection, and manuscript editing. All authors have read and approved the final manuscript.
